# Diagnostic accuracy of synovial chondromatosis of the temporomandibular joint on magnetic resonance imaging

**DOI:** 10.1371/journal.pone.0209739

**Published:** 2019-01-03

**Authors:** Xiaohan Liu, Shaonan Wan, Pei Shen, Yating Qiu, Manoj Kumar Sah, Ahmed Abdelrehem, Minjie Chen, Shanyong Zhang, Chi Yang

**Affiliations:** 1 College of Stomatology, Shanghai Jiao Tong University School of Medicine, Shanghai, China; 2 Department of Oral Surgery, Shanghai Ninth People’s Hospital, Shanghai Jiao Tong University School of Medicine, Shanghai, China; 3 Department of Craniomaxillofacial and Plastic Surgery, Faculty of Dentistry, Alexandria University, Alexandria, Egypt; 4 Shanghai Key Laboratory of Stomatology & Shanghai Research Institute of Stomatology; National Clinical Research Center of Stomatology, Shanghai, China; Yonsei University, REPUBLIC OF KOREA

## Abstract

The purpose of this study was to evaluate the diagnostic accuracy of magnetic resonance imaging (MRI) for synovial chondromatosis (SC) of the temporomandibular joint (TMJ). In this study, 1415 patients (2109 joints) with temporomandibular joint disorders were collected between January 2012 and January 2017. All patients had a preoperative MRI examination and were treated by either arthroscopy or open surgery. On reviewing all MRI images, the number of “positive”, “suspicious”, and “negative” cases was collected afterwards, then the number of reported SC cases in operative data was recorded. The SPSS software was used to process all collected data. The kappa coefficient and ROC curve (AUC-index) with sensitivity and specificity were calculated to evaluate the consistency between MRI and arthroscopy/open surgery. Compared to 156 joints with SC detected by arthroscopy and open surgery, the results of MRI examination showed “positive” in 117 joints, and “negative” in 1938 joints. The number of “true positive”, and “true negative” cases was 95, and 1897 respectively. The AUC-index was 0.86 (0.82–0.90) with a kappa coefficient of 0.74 (P < 0.05). In conclusion, the incidence of synovial chondromatosis diagnosed on MRI was in accordance with the arthroscopic and open surgery. Therefore, being a relatively non-invasive tool, MRI could be recommended as an effective diagnostic modality for SC.

## Introduction

Synovial chondromatosis (SC), a disease entity characterized by the formation of osteocartilaginous bodies and cartilaginous nodules within the synovial membrane, is considered a cartilaginous metaplasia occurring in the synovial joints [1, 2]. Although the majority of SC affects larger joints, it is the most common tumor-like lesion in the temporomandibular joint (TMJ) [1].

For years, surgical approaches (arthroscopy or open surgery) have long been considered as the gold standard for the diagnosis of SC. Based on biopsy specimens, osteocartilaginous nodules with chronic synovitis, and patchy calcifications within a capsule of synovial membrane were detected [3]. Dating back to 1980s, magnetic resonance imaging (MRI), a non-invasive imaging technique, was first reported as a possible investigation method for SC [4]. Nowadays, with the great improvement of resolution and sensitivity, MRI has gradually become an integral part of the standard examination protocol of the TMJ.

According to Massereau et al.,[3] MRI performed well in the diagnosis of osteochondromas with a precise localization ability. However, there are no studies reporting the diagnostic accuracy of MRI in the diagnosis of SC so far. And therefore, the purpose of our study was to evaluate the effectiveness and diagnostic accuracy of magnetic resonance imaging examination for synovial chondromatosis in regards to the gold standard of arthroscopy and open surgery [5, 6].

## Methods

A retrospective cohort study (S1 Checklist) was undertaken between January 2012 and January 2017 in Oral Surgery department, Ninth People’s Hospital, Shanghai Jiao Tong University. The study protocol was reviewed and approved by the Ethics Committee of Shanghai Jiao Tong University School of Medicine and in accordance with the approved guidelines and regulations. All participants (including parents or guardians of the minors who were under the age of 18) provided an informed written consent prior to participation.

### Patients

Patients with symptomatic TMJ disorders had a pre-operative assessment including clinical measurements (maximal interincisal opening, occlusal relationship, and palpation tenderness) and magnetic resonance imaging. Then, patients with chronic pain or dysfunction and had no response to conventional non-surgical treatment (massage therapy, acupuncture, pharmacotherapy, splint therapy, and etc.) were included in this study. The invasive treatment including arthroscopy or open surgery was then performed according to Wilkes’s classification [7, 8].

### MRI examination

According to the MRI protocol, all scans were done at the same clinic by technicians with more than 15 years of experience in medical imaging. General Electric MRI system in 1.5-T/3.0-T (Sign, Milwaukee, WI) was used with a dual 3-inch TMJ surface recoil. T1-weighted images (T1W1) in the oblique sagittal plane were taken at the closed-mouth position. T2-weighted images (T2W2) in the oblique sagittal and coronal planes were taken at the open-mouth position. The parameters of proton density-weighted images (PD) were 2.5-mm-section of thickness, 12-cm field of view, 1800 ms of repetition time (TR), 20 ms of echo time (TE), and 512 * 256 pixels of scanning matrix.

**Fig 1:**
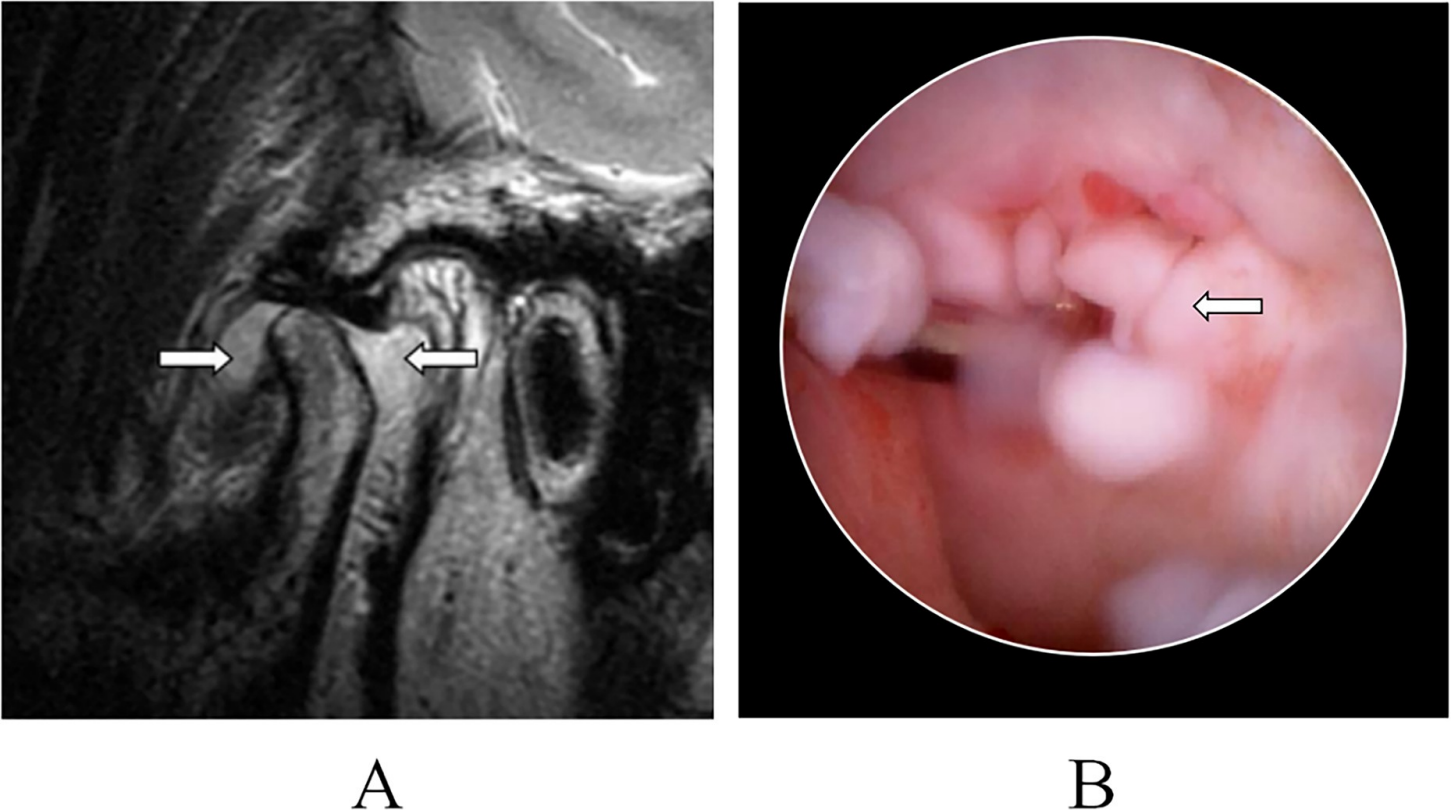
Synovial chondromatosis of temporomandibular joint: Hypointense loose bodies surrounded by hyperintense joint fluid (arrow) was detected on a T2 sequence of MRI, A; Isolated chondrified loose bodies (arrow) was detected during arthroscopic surgery, B.

**Fig 2:**
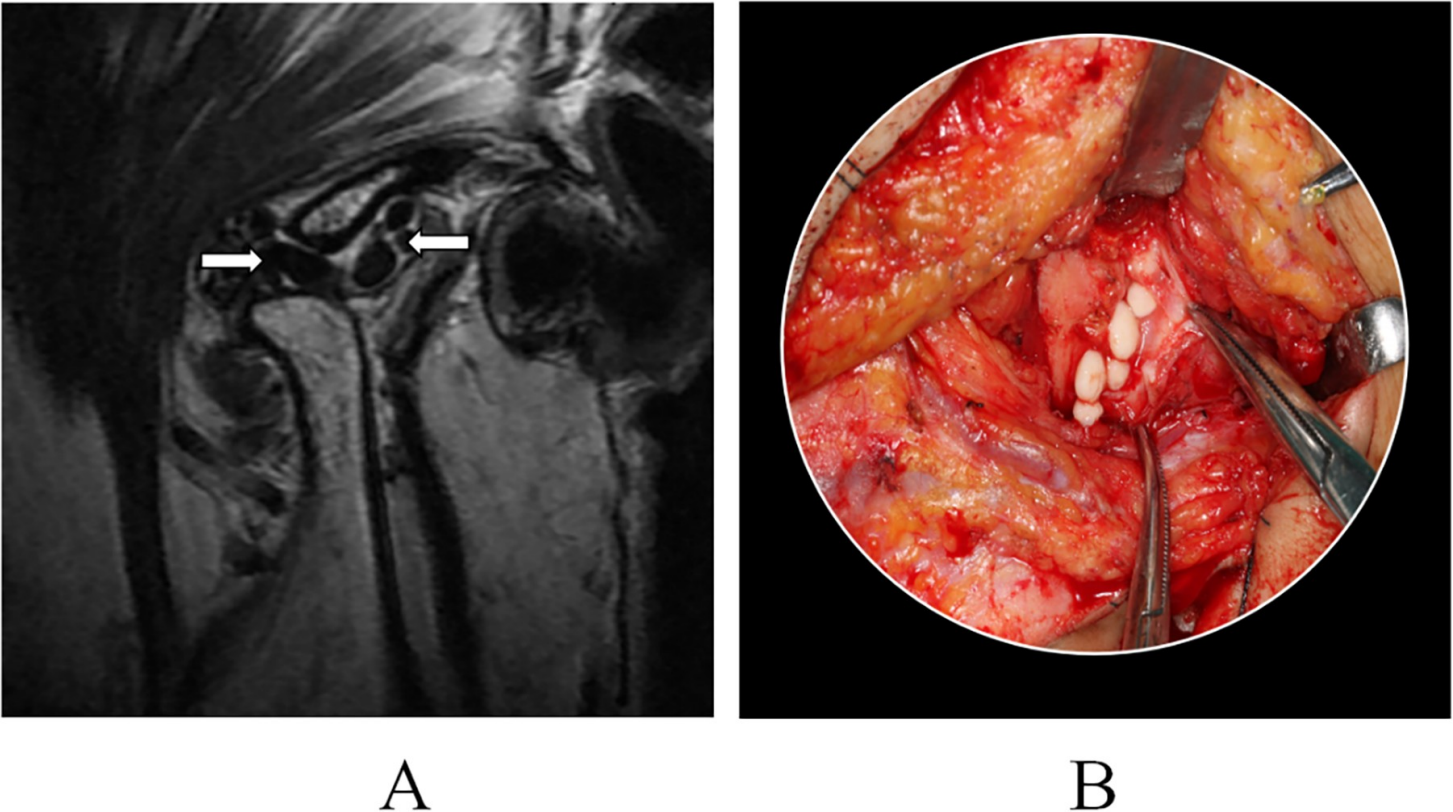
Synovial chondromatosis of temporomandibular joint: Homogeneous mass with chondrified fragments (arrow) was detected on a T2 sequence of MRI, A; Gross specimen was detected during open surgery, B.

**Fig 3:**
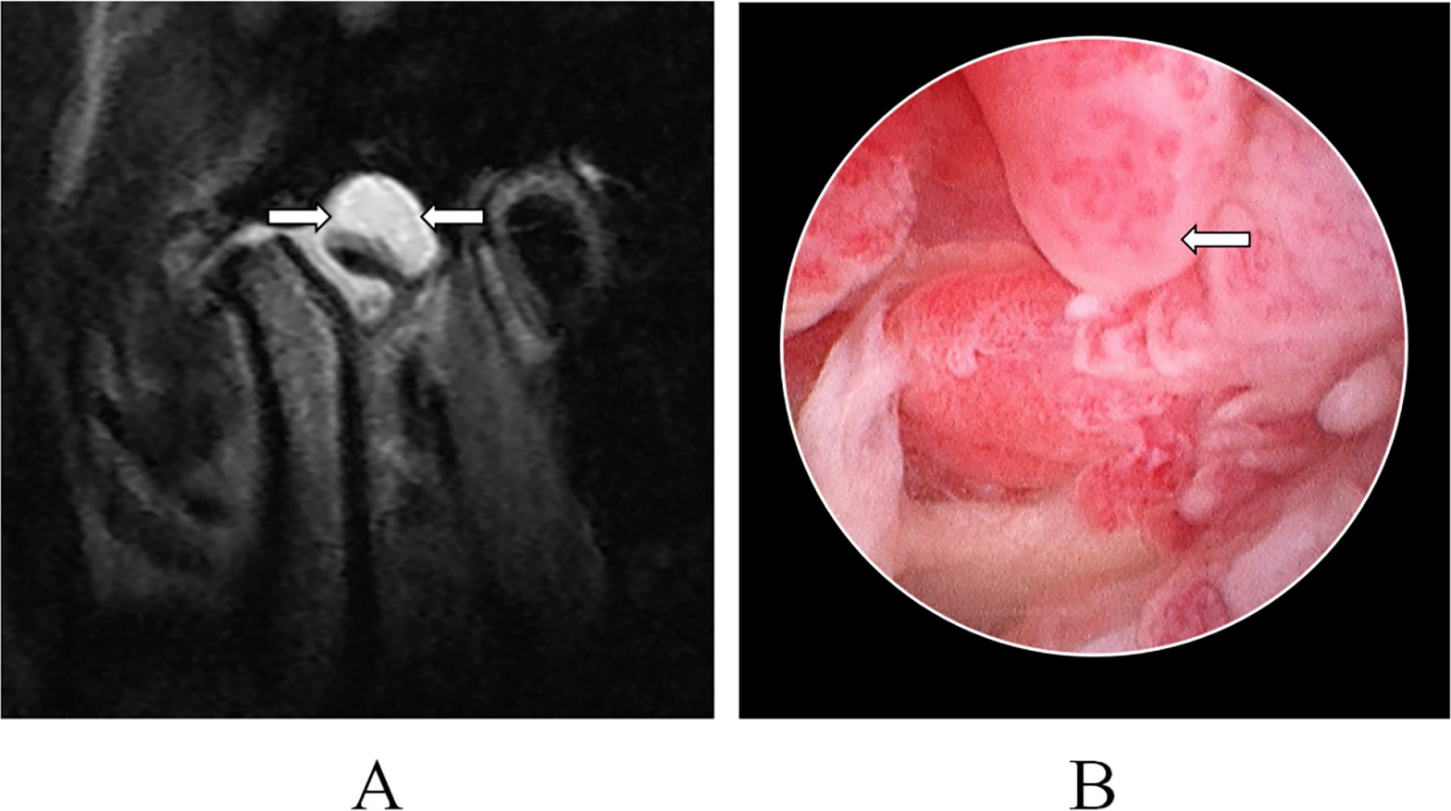
Synovitis of temporomandibular joint: hyperintense synovial fluid was uniformity and moderate signal mass (arrow) was observed on a T2 sequence of MRI, A; No loose body was detected during arthroscopic surgery, B.

The evaluation of lesions in the glenoid fossa on MRI was described as follows: “positive”, when hypointense loose bodies and hyperintense joint fluid (with or without homogeneous mass) were detected on T2-weighted sequence; “suspicious”, when cartilaginous loose bodies are not ossified or calcified yet, but an abnormal large amount of synovial fluid was collected and detected on T2-weighted images with capsular expansion [9]; “negative”, when none of the aforementioned items were found [1, 7, 10, 11] (Figs 1–3).

All images were reviewed blindly in parallel by two independent investigators (XH.L and P.S) according to the criteria. if there was no consensus, then a consistency was achieved through a discussion with a third investigator (C.Y, with more than 30 years of experience in Oral & Maxillofacial Surgery-TMJ).

### Invasive treatment

Either arthroscopy or open surgery was performed as the gold standard for diagnosis of SC in patients following clinical and MRI examinations [12]. Based on the final histopathology, osteocartilaginous nodules with chronic synovitis, and patchy calcifications within a capsule of synovial membrane were detected within the biopsy specimen.

### Statistical analysis

The collected data were processed by using SPSS software, version 21.0 (IBM SPSS Institute Inc, Chicago, USA). A kappa value (kappa coefficient of agreement, 0.21–0.40: fair, 0.41–0.60: moderate, 0.61–0.80 substantial, 0.81–1.00 almost perfect) was calculated to detect the concordance between MRI and arthroscopy/open surgery. The ROC curve with AUC-index was measured to evaluate the diagnostic accuracy of MRI (AUC, <0.50: fail, 0.50–0.70: fair, 0.70–0.90: good; 0.90–1.00: excellent.), which was calculated as follows; Diagnostic accuracy = (true positive + true negative) / (true positive + true negative + false positive + false negative). Probability value under 0.05 (P<0.05) was considered as a statistically significant.

## Results

1415 patients with 2109 joints fulfilling the inclusion criteria were included in this non-randomized, retrospective study with a gender predilection of 1:2.32 (male: 426, female: 989).

**Fig 4:**
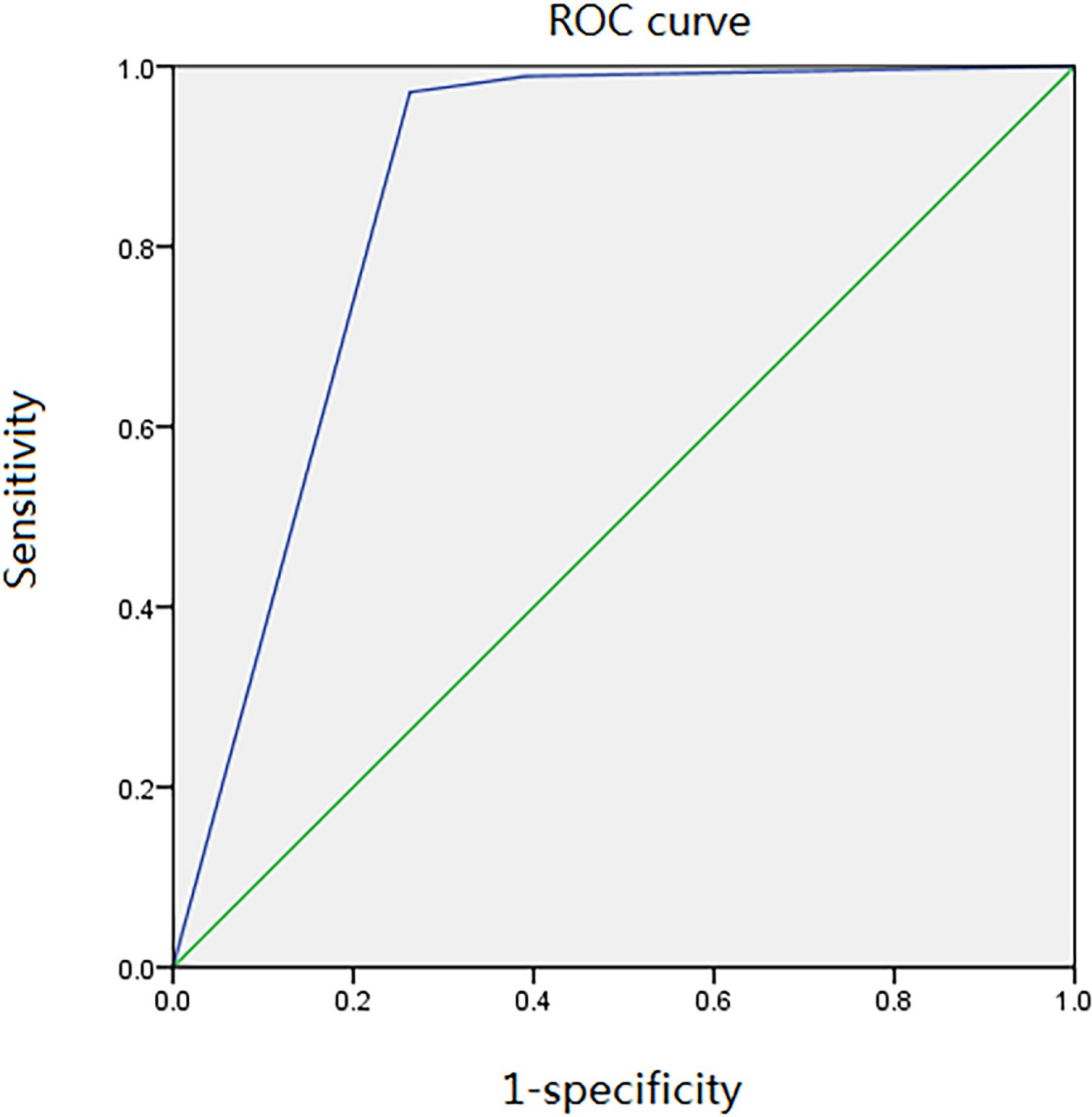
ROC Curve of MRI and arthroscope/open surgery.

Their age ranged from 17 to 65 years, with a mean of 43±6.53 years. There were 721 (50.95%) cases presenting with unilateral side affection and 694 (49.05%) with bilateral involvement. The period of follow-up varied from 0.5 to 14 years (mean 20.12±16.19 months) (Table 1). After the evaluation of MRI, 117 joints were considered as SC “positive”, 54 as “suspicious”, and 1938 as “negative”. Reviewing the operative data after the arthroscopy or open surgery, 156 joints (156/2109) were diagnosed as SC. Comparing the given results of both MRI and arthroscopy or open surgery, “true positive” were found in 95 joints and “false positive” in 22 joints. In the “suspicious” column, 20 joints were “positive” and 34 were “negative” after the invasive treatment. Within the 1938 negative joints, “true negative” was confirmed in 1897 joints and “false negative” in 41 joints (Table 2). The AUC-index for ROC curve was 0.86 (0.82, 0.90) and the concordance agreement was 0.74 (P < 0.05) (Fig 4). Sensitivity, specificity and diagnostic accuracy of the four cut-off points were shown in Table 3.

**Table 1:** Summary of patient characteristics.

Characteristic	Number of patients (n)	%
Gender		
Female	989	69.89
Male	426	30.11
Mean age, year	43±6.53	/
Range	17–65	/
Side		
Unilateral	721	50.95
Bilateral	694	49.05
Mean duration of follow-up, month	20.12±16.19	/
Range	6–168	/

**Table 2:** Outcomes for MRI examination and arthroscopic/open surgery.

		Magnetic resonance imaging			Total
		Positive	Suspicious	Negative	
Arthroscopic/Open surgery	Positive	95	20	41	156
	Negative	22	34	1897	1953
Total		117	54	1938	2109

**Table 3:** Sensitivity, specificity, and diagnostic accuracy.

Cut-off point	Sensitivity (%)	Specificity (%)	Diagnostic accuracy (%)
<1	0.00	100.00	92.60
≥1	60.90	98.89	96.06
≥2	73.72	97.13	75.42
≥3	100.00	0.00	7.34

## Discussion

Synovial chondromatosis (SC) in the temporomandibular joint (TMJ) which was first introduced by Axhausen in 1933 is a benign, tumorous condition [13, 14]. However, the pathogenesis of SC still remains unknown, and the correlative factors including trauma, parafunction, infection and embryologic disturbance are considered as potential risks [2, 15, 16]. Clinically, over 86% of patients complain of symptoms such as joint noise, preauricular pain, and functional limitations. According to previous researches, surgical approaches including arthroscopy and open surgery have been described as the gold standard methods for diagnosis of various diseases including synovial chondromatosis, myositis ossificans, chondrosarcoma, and osteosarcoma, however, still accused of being invasive procedures [17, 18]. Later, magnetic resonance imaging (MRI)-a non-invasive imaging tool which demonstrated the typical articular calcifications in a “ring-and-arc” feature was reported to be pathognomonic (70%–95%) in large articulations such as knee, hip, and ankle joints [19–22]. According to Kim et al., [23] features of primary synovial chondromatosis including synovial hyperintensity (87%), bone erosion (73%), and homogeneous conglomeration (73%) of the hip lesions, were recognized in 15 cases based on MRI examination. Meanwhile, MRI examination was also reported to be a superior diagnostic tool for SC in the TMJ [24, 25]. In Ardekian et al.’s study [26], pathological cartilaginous changes were accurately detected in MRI examinations of 10 out of 11 patients. However, Santler et al., [27] argued that it was difficult to distinguish structures such as cartilage, cortical bone or synovial fluid, which usually presented with a similar manifestation of low signal-density on T1-weighted sequence. In our study, 117 joints (out of 2109 joints) were diagnosed as SC “positive” based on a preoperative MRI examination. Later, SC was confirmed in 156 joints (out of 2109 joints) during the process of surgical treatment. Accordingly, the estimated diagnostic accuracy of MRI in SC was therefore nearly 96.06% with a substantial kappa coefficient and a good ROC curve. Meanwhile, the “false positive” was reported in 22 joints. Among which, 19 synovitis cases with disc displacements were firstly misdiagnosed as SC on MRI examination, because the observers misinterpreted celluloses as loose bodies. Furthermore, 3 cases finally came to be pigmented villonodular synovitis (PVNS). According to the pathology findings, small particles detected on MRI examination were found to be hemochromatosis spots immersed in the joint fluid. Among the 54 “suspicious” joints with hyperintense synovial fluid alone (12 with moderate signal mass) on MRI, fibrous cord hyperplasia and small nodular calcifications were observed in 20 joints during surgery. The remaining 34 cases turned out to be synovitis with disc displacement. According to Leibur et al., over 17% (5/29) of SC was diagnosed in patients treated by arthroscopy for TMJ internal derangement [28]. In our study, the 41 “false negative” joints, in which neither hypointense loose bodies nor hyperintense joint fluid were detected on T2-weighted sequence, except for variable enlargement of the joint capsule and anterior disc displacement without reduction, were reported later and proved as under-diagnosed. The findings of MRI examination which to a certain extent depend on the degree of mineralization, are sometimes variable. In some early cases, especially when lesions are uniformly distributed and no shaped loose bodies or hyperintense joint fluid were evident on T2-weighted sequence, SC might not be easily detected on MRI examination. Moreover, the under-diagnosed cases could be explained by the occurrence of few small-sized hypointense loose bodies hiding within the hyperintense joint fluid (T2-weighted sequence). Therefore, further researches such as contrast-enhanced computed tomography (CT) or arthrography are recommended in order to accurately diagnose the early stage of SC.

To our knowledge, this is the first article evaluating the accuracy of MRI examination in diagnosis of synovial chondromatosis of the TMJ. The study analysis was based on a large database with long-term follow-up, therefore, more valid outcomes. In order to enhance the accuracy and reliability of the findings, both data extraction and statistical analysis were all performed by two investigators independently with the sensitivity and specificity calculated. The results showed that MRI would be helpful as a reliable diagnostic tool adding to the operative confirmation of synovial chondromatosis. While further researches combined by clinical, imaging, and histologic examinations are still needed in future investigations.

In conclusion, considering the good diagnostic accuracy, we recommend magnetic resonance imaging (MRI) as a relatively non-invasive and effective diagnostic modality in detecting synovial chondromatosis.

## Supporting information

S1 Checklist (DOCX)
