# Supplementary material for: Diagnostic accuracy of synovial chondromatosis of the temporomandibular joint on magnetic resonance imaging
Source: PLoS One. 2019 Jan 3;14(1):e0209739. doi: 10.1371/journal.pone.0209739 (PMC6317805; doi:10.1371/journal.pone.0209739)
Supplement: S1 Checklist — (DOCX) [file pone.0209739.s001.docx]

STROBE Statement—checklist of items that should be included in reports of observational studies

|  | Item No. | Recommendation | Page  No. | Relevant text from manuscript |
| --- | --- | --- | --- | --- |
| **Title and abstract** | 1 | (*a*) Indicate the study’s design with a commonly used term in the title or the abstract | 2 | In this study, 1415 patients (2109 joints) of temporomandibular joint disorder (TMD) were collected between January 2012 and January 2017. |
|  |  | (*b*) Provide in the abstract an informative and balanced summary of what was done and what was found | 2 | The purpose of this study was to evaluate the …… could be recommended as an effective diagnose method of SC. |
| Introduction | | | |  |
| Background/rationale | 2 | Explain the scientific background and rationale for the investigation being reported | 3 | For many years, surgical approaches (arthroscopy or open surgery) have long been performed…… regarding to the gold standard of arthroscopy and open surgery. |
| Objectives | 3 | State specific objectives, including any prespecified hypotheses | 3 | For many years, surgical approaches (arthroscopy or open surgery) have long been performed…… regarding to the gold standard of arthroscopy and open surgery. |
| Methods | | | |  |
| Study design | 4 | Present key elements of study design early in the paper | 3 | A retrospective cohort study was undertaken between January 2012 and January 2017 at the TMJ department of the Ninth People’s Hospital, Shanghai Jiao Tong University. |
| Setting | 5 | Describe the setting, locations, and relevant dates, including periods of recruitment, exposure, follow-up, and data collection | 3 | A retrospective cohort study was undertaken between January 2012 and January 2017 at the TMJ department of the Ninth People’s Hospital, Shanghai Jiao Tong University. |
| Participants | 6 | (*a*) *Cohort study*—Give the eligibility criteria, and the sources and methods of selection of participants. Describe methods of follow-up  *Case-control study*—Give the eligibility criteria, and the sources and methods of case ascertainment and control selection. Give the rationale for the choice of cases and controls  *Cross-sectional study*—Give the eligibility criteria, and the sources and methods of selection of participants | 3 | Patients with symptomatic TMJ disorders had a pre-operative manifestations ......performed according to Wilkes’s classification. |
|  |  | (*b*) *Cohort study*—For matched studies, give matching criteria and number of exposed and unexposed  *Case-control study*—For matched studies, give matching criteria and the number of controls per case | 3 | Patients with symptomatic TMJ disorders had a pre-operative manifestations ......performed according to Wilkes’s classification. |
| Variables | 7 | Clearly define all outcomes, exposures, predictors, potential confounders, and effect modifiers. Give diagnostic criteria, if applicable | 4 | The evaluation of lesions in the fossa on MRI was described as follows: “positive”, when hypointense loose bodies with hyperintense joint fluid (T2-weighted sequence)…… aforementioned items were found (Figs. 1-3). |
| Data sources/ measurement | 8* | For each variable of interest, give sources of data and details of methods of assessment (measurement). Describe comparability of assessment methods if there is more than one group | 4 | The evaluation of lesions in the fossa on MRI was described as follows: “positive”, when hypointense loose bodies with hyperintense joint fluid (T2-weighted sequence)…… aforementioned items were found (Figs. 1-3). |
| Bias | 9 | Describe any efforts to address potential sources of bias | 4 | All images were reviewed blindly in parallel by two independent reviewers (XH.L and P.S) according to the criteria. Any discrepancy was resolved through discussion with a third investigator (C.Y, with more than 30 years of experience in medical TMJ) |
| Study size | 10 | Explain how the study size was arrived at | 3 | A retrospective cohort study was undertaken between January 2012 and January 2017 at the TMJ department of the Ninth People’s Hospital, Shanghai Jiao Tong University. |

Continued on next page

| Quantitative variables | 11 | Explain how quantitative variables were handled in the analyses. If applicable, describe which groupings were chosen and why | 4 | The collected data were processed by using SPSS …… Probability value under 0.05 (P<0.05) was considered as statistically significant. |
| --- | --- | --- | --- | --- |
| Statistical methods | 12 | (*a*) Describe all statistical methods, including those used to control for confounding | 3,4 | Patients with symptomatic TMJ disorders had a pre-operative manifestations ......performed according to Wilkes’s classification. |
|  |  | (*b*) Describe any methods used to examine subgroups and interactions | N/A | N/A |
|  |  | (*c*) Explain how missing data were addressed | 3,4 | Patients with symptomatic TMJ disorders had a pre-operative manifestations ......performed according to Wilkes’s classification. |
|  |  | (*d*) *Cohort study*—If applicable, explain how loss to follow-up was addressed  *Case-control study*—If applicable, explain how matching of cases and controls was addressed  *Cross-sectional study*—If applicable, describe analytical methods taking account of sampling strategy | 3,4 | Patients with symptomatic TMJ disorders had a pre-operative manifestations ......performed according to Wilkes’s classification. |
|  |  | (*e*) Describe any sensitivity analyses | 4 | The collected data were processed by using SPSS® …… Probability value under 0.05 (P<0.05) was considered as statistically significant. |
| Results | | | | |
| Participants | 13* | (a) Report numbers of individuals at each stage of study—eg numbers potentially eligible, examined for eligibility, confirmed eligible, included in the study, completing follow-up, and analysed | 4,5 | 1415 patients met the inclusion criteria were included in retrospective ……Sensitivity, specificity and diagnostic accuracy of the four cut-off points were shown in Table 2B. |
|  |  | (b) Give reasons for non-participation at each stage | 4,5 | 1415 patients met the inclusion criteria were included in retrospective ……Sensitivity, specificity and diagnostic accuracy of the four cut-off points were shown in Table 2B. |
|  |  | (c) Consider use of a flow diagram | N/A | N/A |
| Descriptive data | 14* | (a) Give characteristics of study participants (eg demographic, clinical, social) and information on exposures and potential confounders | 4,5 | 1415 patients met the inclusion criteria were included in retrospective ……Sensitivity, specificity and diagnostic accuracy of the four cut-off points were shown in Table 2B. |
|  |  | (b) Indicate number of participants with missing data for each variable of interest | 4,5 | 1415 patients met the inclusion criteria were included in retrospective ……Sensitivity, specificity and diagnostic accuracy of the four cut-off points were shown in Table 2B. |
|  |  | (c) *Cohort study*—Summarise follow-up time (eg, average and total amount) | 4,5 | 1415 patients met the inclusion criteria were included in retrospective ……Sensitivity, specificity and diagnostic accuracy of the four cut-off points were shown in Table 2B. |
| Outcome data | 15* | *Cohort study*—Report numbers of outcome events or summary measures over time | 4,5 | 1415 patients met the inclusion criteria were included in retrospective ……Sensitivity, specificity and diagnostic accuracy of the four cut-off points were shown in Table 2B. |
|  |  | *Case-control study—*Report numbers in each exposure category, or summary measures of exposure |  |  |
|  |  | *Cross-sectional study—*Report numbers of outcome events or summary measures |  |  |
| Main results | 16 | (*a*) Give unadjusted estimates and, if applicable, confounder-adjusted estimates and their precision (eg, 95% confidence interval). Make clear which confounders were adjusted for and why they were included | 4,5 | 1415 patients met the inclusion criteria were included in retrospective ……Sensitivity, specificity and diagnostic accuracy of the four cut-off points were shown in Table 2B. |
|  |  | (*b*) Report category boundaries when continuous variables were categorized | 4,5 | 1415 patients met the inclusion criteria were included in retrospective ……Sensitivity, specificity and diagnostic accuracy of the four cut-off points were shown in Table 2B. |
|  |  | (*c*) If relevant, consider translating estimates of relative risk into absolute risk for a meaningful time period | 4,5 | 1415 patients met the inclusion criteria were included in retrospective ……Sensitivity, specificity and diagnostic accuracy of the four cut-off points were shown in Table 2B. |

Continued on next page

| Other analyses | 17 | Report other analyses done—eg analyses of subgroups and interactions, and sensitivity analyses | 4,5 | 1415 patients met the inclusion criteria were included in retrospective ……Sensitivity, specificity and diagnostic accuracy of the four cut-off points were shown in Table 2B. |
| --- | --- | --- | --- | --- |
| Discussion | | | | |
| Key results | 18 | Summarise key results with reference to study objectives | 5,6 | In this study, 117 joints were diagnosed as SC “positive” after a preoperative MRI examination. …… recommended in order to investigate in magnetic resonance imaging of early synovial chondromatosis. |
| Limitations | 19 | Discuss limitations of the study, taking into account sources of potential bias or imprecision. Discuss both direction and magnitude of any potential bias | 6 | In this study, 117 joints were diagnosed as SC “positive” after a preoperative MRI examination. …… recommended in order to investigate in magnetic resonance imaging of early synovial chondromatosis. |
| Interpretation | 20 | Give a cautious overall interpretation of results considering objectives, limitations, multiplicity of analyses, results from similar studies, and other relevant evidence | 6 | To our knowledge, this is the first article assessed the accuracy of MRI examination in diagnosis of …… further researches combined by clinical, imaging, and histologic examination are still needed in future investigations. |
| Generalisability | 21 | Discuss the generalisability (external validity) of the study results | 6 | In conclusion, given to the good diagnostic accuracy, we recommended magnetic resonance imaging (MRI) as a relative non-invasive and effective diagnostic modality in detecting synovial chondromatosis. |
| Other information | |  | | |
| Funding | 22 | Give the source of funding and the role of the funders for the present study and, if applicable, for the original study on which the present article is based | 6,7 | This study was supported by the Youth Program of National Natural Scienc…… funding sources had no role in the study design, collection, analysis, or interpretation of data, or writing of the report. |

*Give information separately for cases and controls in case-control studies and, if applicable, for exposed and unexposed groups in cohort and cross-sectional studies.

**Note:** An Explanation and Elaboration article discusses each checklist item and gives methodological background and published examples of transparent reporting. The STROBE checklist is best used in conjunction with this article (freely available on the Web sites of PLoS Medicine at http://www.plosmedicine.org/, Annals of Internal Medicine at http://www.annals.org/, and Epidemiology at http://www.epidem.com/). Information on the STROBE Initiative is available at www.strobe-statement.org.
